# Systematic and Comprehensive Automated Ventricle Segmentation on Ventricle Images of the Elderly Patients: A Retrospective Study

**DOI:** 10.3389/fnagi.2020.618538

**Published:** 2020-12-16

**Authors:** Xi Zhou, Qinghao Ye, Yinghui Jiang, Minhao Wang, Zhangming Niu, Wade Menpes-Smith, Evandro Fei Fang, Zhi Liu, Jun Xia, Guang Yang

**Affiliations:** ^1^Department of Radiology, Shenzhen Second People's Hospital, The First Affiliated Hospital of Shenzhen University Health Science Center, Shenzhen, China; ^2^Hangzhou Ocean's Smart Boya Co., Ltd., Hangzhou, China; ^3^Mind Rank Ltd., Hongkong, China; ^4^Aladdin Healthcare Technologies Ltd., London, United Kingdom; ^5^Department of Clinical Molecular Biology, University of Oslo, Oslo, Norway; ^6^School of Information Science and Engineering, Shandong University, Qingdao, China; ^7^Cardiovascular Research Centre, Royal Brompton Hospital, London, United Kingdom; ^8^National Heart and Lung Institute, Imperial College London, London, United Kingdom

**Keywords:** deep learning, neuroimage, magnetic resonance imaging, ventricular segmentation, image segmentation, convolutional neural network (CNN), computer tomography (CT)

## Abstract

**Background and Objective:** Ventricle volume is closely related to hydrocephalus, brain atrophy, Alzheimer's, Parkinson's syndrome, and other diseases. To accurately measure the volume of the ventricles for elderly patients, we use deep learning to establish a systematic and comprehensive automated ventricle segmentation framework.

**Methods:** The study participation included 20 normal elderly people, 20 patients with cerebral atrophy, 64 patients with normal pressure hydrocephalus, and 51 patients with acquired hydrocephalus. Second, get their imaging data through the picture archiving and communication systems (PACS) system. Then use ITK software to manually label participants' ventricular structures. Finally, extract imaging features through machine learning.

**Results:** This automated ventricle segmentation method can be applied not only to CT and MRI images but also to images with different scan slice thicknesses. More importantly, it produces excellent segmentation results (Dice > 0.9).

**Conclusion:** This automated ventricle segmentation method has wide applicability and clinical practicability. It can help clinicians find early disease, diagnose disease, understand the patient's disease progression, and evaluate the patient's treatment effect.

## Introduction

The volume of the ventricle has always been closely related to degenerative brain diseases and traumatic brain injury. Researchers have also described that enlargement of the ventricles is an important characteristic of medical conditions such as schizophrenia, Parkinson's disease, Alzheimer's disease, hydrocephalus, and trauma to the brain (Silbert et al., 2003; Thompson et al., 2004; Chou et al., 2009; Liu et al., 2010; Cavedo et al., 2012; Khan et al., 2012; Anandh et al., 2016; Del et al., 2016; Owen et al., 2018; Kocaman et al., 2019; Lundervold et al., 2019). In some disease diagnosis guidelines, EI > 0.3 is often defined as ventricular enlargement (Relkin et al., 2005; Mori et al., 2012). However, some studies have shown that the correlation between EI and ventricle volume is only 0.619 (Toma et al., 2011). The measurement of EI is affected by different scan baselines and different measurement planes, which only reflects the local conditions of the ventricle at the selected level, and cannot fully assess the size of the ventricle (Ambarki et al., 2010). Moreover, EI is sensitive to the expansion of the ventricle to both sides, and the effect is not good when evaluating patients whose ventricle expands to the long axis (He et al., 2020). At the same time, in the normal elderly, the range of EI is relatively wide. Taking EI = 0.3 as the cut-off value, it is difficult to effectively distinguish between normal and enlarged ventricles (Brix et al., 2017). Therefore, when we need the volume of the ventricle, and the volume of the ventricle is measurable, then we should use it (Ambarki et al., 2010). Because of this, research on ventricle segmentation methods has brought much attention, and researchers have continuously optimized algorithms to make better and more accurate estimation (Chen et al., 2009; Coupe et al., 2011; Kempton et al., 2011; Poh et al., 2012; Qiu et al., 2015; Tang et al., 2015, 2018; Qian et al., 2017; Cherukuri et al., 2018; Shao et al., 2019; Dubost et al., 2020).

Volumetric measurement is the only method to directly determine the ventricular size. It is realized by segmentation, which can be roughly categorized into automated segmentation and manual segmentation (Huff et al., 2019). The manual segmentation technique is the gold standard for volumetric quantification of regional brain structures (Kocaman et al., 2019), but when dealing with more data, manual segmentation of the ventricles is time-consuming, subjective, and less reproducible (Chou et al., 2008; Liu et al., 2009; Poh et al., 2012). Therefore, it is highly in demand for an automated ventricle segmentation method to be developed and machine and deep learning based methods have emerged as the new era.

In the previous automated ventricle segmentation methods, researchers often conducted single-mode studies, i.e., segment either on CT images (Liu et al., 2010; Poh et al., 2012; Qian et al., 2017; Cherukuri et al., 2018) or MRI images (Qiu et al., 2015; Tang et al., 2015, 2018). Therefore, the developed automated ventricle segmentation methods were rarely interchangeable. Moreover, various algorithms might perform differently in segmenting different sections of the ventricles (Chen et al., 2009; Coupe et al., 2011; Shao et al., 2019; Dubost et al., 2020). Most previous machine learning (including deep learning) based studies were developed using images with a slice thickness of <3 mm, because at the same scanning distance, the smaller of the image thickness, the more images could be obtained, which could be more conducive for machine/deep learning algorithms to extract more image features (Xia et al., 2004; Coupe et al., 2011; Kempton et al., 2011). However, in clinical practice, due to time constraints, images with larger slice thicknesses are more common. Therefore, the clinical usage of these methods is relatively limited.

The reproducibility of machine/deep learning based algorithms across different scanners and pulse sequences had not always been comprehensively examined (Kempton et al., 2011). Moreover, their accuracy in different clinical populations and sensitivity to real changes in brain volume could still be improved. A larger slice thickness would increase the partial volume effect, which could have a significant negative impact on the algorithm accuracy. For example, the intraventricular calcified area located at the border of the ventricle may not be recognized. Some cerebellar ventricle areas (anterior, posterior, and inferior horns of the lateral ventricle) may not be recognized because they are not connected to the core of the lateral ventricle (Liu et al., 2010). In some automated ventricle segmentation methods, pathological ventricles were not included (Huff et al., 2019), but pathological ventricles are common in the elderly, especially in patients with acquired hydrocephalus, because they may have brain trauma, brain tumors, subarachnoid hemorrhage, and it becomes extremely difficult to delineate the ventricle from these patients. Previous literature also reported the segmentation of the ventricle of idiopathic Normal Pressure Hydrocephalus (iNPH) patients (Shao et al., 2019). These patients are prone to segmentation failure due to the enlarged ventricle. Therefore, our purpose is not only to optimize the algorithm and obtain more accurate results but more importantly, to make this automated ventricle segmentation method be more widely used and be trustworthy for clinical practice.

In summary, the goal of this study is to establish a deep learning based automated ventricle segmentation method that can be generally used for both CT and MRI images, and is versatile for both thin-layer and thick-layer images.

## Methods

### Participants

**Figure 1:**
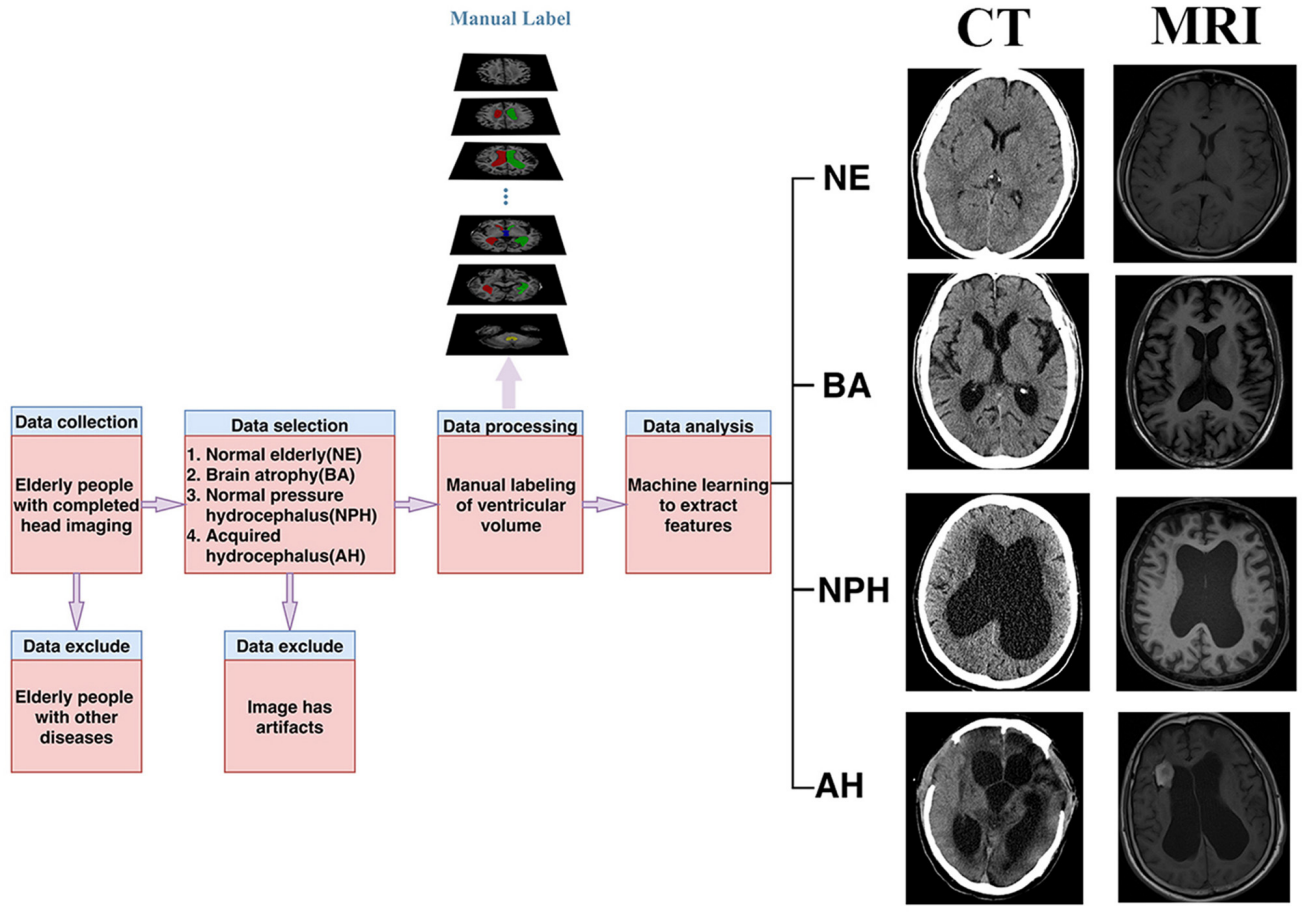
Study flow chart for the inclusion of participants.

First, we selected the images of patients over 60 years old who underwent brain CT or MRI examinations at Shenzhen Second People's Hospital from January 1, 2016 to December 31, 2019. Second, as we aimed to delineate the ventricle and perform a comprehensive analysis, we chose the normal elderly, the elderly with brain atrophy, the elderly with idiopathic normal pressure hydrocephalus, and the elderly with acquired hydrocephalus people. Because the shape and size of the ventricles of these four types of patients are very representative, showing a trend from normal to severe, which can help us systematically and comprehensively analyze the ventricular system. Third, the diagnostic results of these patients were agreed upon by two radiologists with more than 10 years of work experience and strictly followed the disease diagnosis guidelines. Last but not least, due to a large number of normal elderly people and patients with brain atrophy, a large number of manual labeling would be infeasible. However, there is no obvious deformation of their ventricle structure, and it is easier for the automatic ventricle segmentation of the normal elderly and the elderly with brain atrophy. Therefore, we arranged the normal elderly and the elderly with brain atrophy in the order of the time of head imaging examination and numbered them, and made the numbers into small pieces of paper, and placed them in a large carton. Using a simple random sampling method, let 20 doctors in the radiology department randomly sample small pieces of paper. In the end, we randomly selected 20 normal elderly people and elderly people with brain atrophy for manual marking. The flowchart of the admission and exclusion of patients is shown in Figure 1, and the basic study population description is shown in Table 1.

**Table 1:** Demographic information of subjects used in this study.

	**Normal elderly**	**Brain atrophy**	**Normal pressure hydrocephalus**	**Acquired hydrocephalus**
	**(*n* = 20)**	**(*n* = 20)**	**(*n* = 64)**	**(*n* = 51)**
Age* (years)	64.81 ± 3.19	70.95 ± 5.55	70.77 ± 6.81	67.01 ± 5.41
Sex (male:female)	11:9	12:8	37:27	31:20
**Scanning status of imaging equipment**^#^				
CT-1	4	3	19	24
CT-2	6	7	28	16
MRI-1	5	4	23	18
MRI-2	5	6	31	20

* *Age reported as mean ± standard deviation*.

# *CT-1 represents the CT instrument of SOMATOM Definition Flash from Siemens, Germany. CT-2 represents the CT instrument of SOMATOM Emotion 16 from Siemens, Germany. MRI-1 represents the 1.5T MR scanner (Avanto, Siemens, Erlangen, Germany). MRI-2 represents the 3.0T MRI scanner (Prisma, Siemens, Erlangen, Germany). The slice thickness of CT image includes: 0.5, 1.0, 1.5, 2.0, 4.8, 5.0 mm. The slice thickness of MRI image includes: 1.0, 7.8, 8.0 mm*.

### Ethics Statement

This study was carried out in accordance with the recommendations of the Ethics Committee of The First Affiliated Hospital of Shenzhen University and Shenzhen Second People's hospital. All subjects gave written informed consent in accordance with the Declaration of Helsinki.

### Imaging Protocol and Label

First, a CT scan of the head was performed on two CT instruments, one of which was SOMATOM Definition Flash from Siemens, Germany, and the other was SOMATOM Emotion 16 from Siemens, Germany. Secondly, MRI examinations were conducted using a 1.5T MR scanner (Avanto, Siemens, Erlangen, Germany), and a 3.0T MRI scanner (Prisma, Siemens, Erlangen, Germany). All images were stored in the picture archiving and communication systems (PACS).

Then manually delineation of the ventricle was conducted. For MRI images, we chose T1WI for manual labeling. The specific manual labeling process is as follows: (1) two radiologists with 10 years of clinical experience used ITK software to label the ventricles; (2) a senior radiologist with 20 years of clinical experience evaluated the delineation results of the ventricles and made adjustment if inaccurate manual labeling was found; and (3) for the controversial annotated cases, we invited a neurology expert and a neurosurgery expert to discuss, and modifications and the final annotation results were approved by them.

We defined the thick layer image when the scan layer thickness was >3 mm, and otherwise, it was defined as the thin layer image. Therefore, all images were classified into four groups, i.e., thin-slice CT images, thick-slice CT images, thin-slice MRI images, and thick-slice MRI images.

### The Proposed Deep Learning Framework

In real-world scenarios, the thick-slice images are more easily obtained, while thin-slices images are rare, and it is more difficult for clinicians to annotate them. Moreover, the distribution of different image thicknesses can result in the *domain shift* problem that can confuse the deep learning models (Yan et al., 2019). Therefore, we proposed a thickness agnostic image segmentation model, which only required the annotation of thick-slice images for the model training.

Our goal is to utilize the unlabeled thin-slice images to minimize the performance gap between thick-slice and thin-slice images. In our model, the thick-slice images are denoted as $D_{S}=\left\{\left(x_{s},y_{s}\right)|\;x_{s}\in R^{H\times W\times 3},y_{s}\in R^{H\times W}\right\}$, and the thin-slices images are represented as $D_{T}=\left\{x_{t}|x_{t}\in R^{H\times W\times \;3}\right\}$.

**Figure 2:**
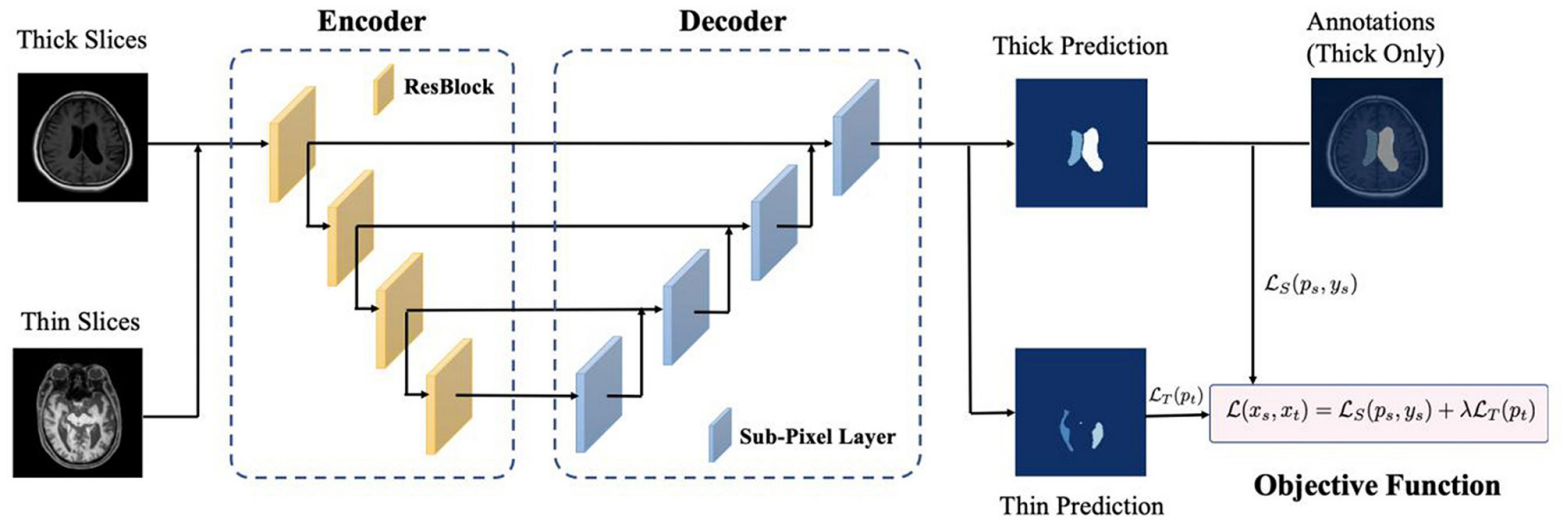
The workflow of proposed methods.

With the increased development and application of deep learning methods, encoder-decoder based architectures (Milletari et al., 2016; Zhou et al., 2018) are widely used in medical image segmentation. The workflow of our proposed deep learning based framework is presented in Figure 2. For image feature extraction and reconstruction, we adopted ResNet-34, which was pre-trained on ImageNet datasets as the encoder of input images. For the decoder, sub-pixel convolution was used for constructing segmentation results since the deconvolution operation was computationally heavy and interpolation-based methods could not bring additional information to improve the segmentation. The sub-pixel convolution can be then represented as

(1) $$\begin{array}{r} F^{L}=SP\left(W_{L}*F^{L-1}+b_{L}\right), \end{array}$$

where *SP*(·) operator transforms and arranges a tensor with the shape of *H* × *W* × *C* × *r*^2^ into a tensor shaped in *rH* × *rW* × *C*, *F*^*L*−1^ and *F*^*L*^ are the input feature and output feature, *W*_*L*_ and *b*_*L*_ are the parameters of the sub-pixel convolution operator.

We took both thick-slice and thin-slice images as the input and optimized our model with the following objective function

(2) $$\begin{array}{r} L\left(x_{s},x_{t}\right)=L_{S}\left(p_{s},y_{s}\right)+\lambda L_{T}\left(p_{t}\right), \end{array}$$

where λ is a hyper-parameter for weighting the impact of *L*_*s*_ and *L*_*T*_, *p*_*s*_ and *p*_*t*_ are the model's predictions of the segmentation probability shaped of *H* × *W* × *C*. *L*_*S*_ is the cross-entropy loss defined as follows

(3) $$\begin{array}{r} L_{S}=-\frac{1}{HW}\overset{HW}{\underset{n=1}{\sum }}\overset{C}{\underset{c=1}{\sum }}y_{s}^{n,c}\log p_{t}^{n,c}. \end{array}$$

Since we expect our model to learn an accurate segmentation paradigm for both thick-slice images and thin-slice images, the *L*_*T*_ can be regarded as the distance between the probability distribution of the target domain (thin-slice domain) *p*_*t*_ and the uniform distribution $U=\frac{1}{C}$. Therefore, minimizing the distance between the two distributions enables classes to be more separable. Because it implicitly pushes the image features away from the decision boundary and makes alignment between two distributions. Mathematically, the objective function of thin-slice images is formulated as

(4) $$\begin{array}{r} L_{T}=-D_{f}\left(p_{t}^{n,c}||U\right)=-\frac{1}{C}\overset{C}{\underset{c=1}{\sum }}f\left(Cp_{t}^{n,c}\right). \end{array}$$

Most existing methods (Vu et al., 2019) would choose *f* (*x*) = *x* log *x*, which is alternatively named as the KL-divergence. However, one of the main obstacles is that when adopting KL-divergence for *L*_*T*_ as the objective function, the gradient of *L*_*T*_ would be extremely imbalanced between easy and hard samples. Taking the binary case as an example, the gradient can be computed as

(5) $$\begin{array}{r} \frac{\partial L_{T}}{\partial p_{t}^{n,i}}=\log\left(1-p_{t}^{n,i}\right)-\log p_{t}^{n,i}, \end{array}$$

of which the increasing speed is faster as $p_{t}^{n,i}$ becomes larger.

Therefore, to mitigate the unbalancing problem represented above, instead of choosing *f* (*x*) = *x* log *x*, we select Pearson χ^2^ divergence [i.e., *f* (*x*) = *x*^2^ − 1] for *L*_*T*_. Therefore, the gradient of *L*_*T*_ can be noted as

(6) $$\begin{array}{r} \frac{\partial L_{T}}{\partial p_{t}^{n,i}}=2-4p_{t}^{n,i}, \end{array}$$

which balances the gradient between easy and hard samples. During model training, the above loss functions were optimized iteratively. For testing, we fed each slice of images as the input and get the predicted segmentation.

## Result

### Diagnostic Efficiency

For experiments, we collected thick-slice and thin-slice samples from iNPH patients with different modalities (MRI and CT) as Table 2 shows. It is of note that we only used the annotations from thick-slice images for our supervised deep learning. We investigated the performance of U-Net (Ronneberger et al., 2015) and U-Net++ (Zhou et al., 2018) on thin-slice and thick-slice images. Both U-Net and U-Net++ adopted encoders and decoders structure while using the middle features to maintain the information of images. As shown in Table 3, compared to conventional and state-of-the-art models, our method achieved significant improvement on both thick-slice and thin-slice images. To further illustrate, our method outperformed U-Net and U-Net++, which are commonly used in medical segmentation, by a large margin. Besides, with the help of a pre-trained ResNet-34 encoder, our model could gain at most 0.1 Dice coefficient on thick-slice images.

**Table 2:** The number of thick-slice and thin-slice images used for our study.

**Modality**	**The number of the training set**		**The number of the testing set**	
	**Thick-slice**	**Thin-slice**	**Thick-slice**	**Thin-slice**
MRI	1,013	1,629	189	982
CT	2,611	2,595	309	492

*For CT and MRI datasets, each dataset is divided into two groups. The training set is only used for model training and optimization, while the testing set is used to validate the effectiveness of the trained model*.

**Table 3:** Comparison results (Dice) of our method vs. other state-of-the-art methods.

**Method**	**MRI**			**CT**		
	**Thick**	**Thin**	**Mixed**	**Thick**	**Thin**	**Mixed**
U-Net (Ronneberger et al., 2015)	0.9226	0.7665	0.8353	0.9351	0.7987	0.8513
U-Net++ (Zhou et al., 2018)	0.9159	0.8495	0.8602	**0.9421**	0.7797	0.8424
Ours	**0.9323**	**0.9056**	**0.9099**	0.9365	**0.8697**	**0.8954**

*For each trained model, we tested it on thick-slice data only, thin-slice data only, and the combination of these two data. We can see that our method outperforms the other two state-of-the-art methods by a large margin, especially on the thin-slice images. Bold values indicate the best performance*.

### Component Analysis

To examine the influence of each component in our method, we conducted ablation studies to verify the effectiveness of our method, and the results are summarized in Table 4. We can observe that if our model only trained on thick-slice images, we can get comparable results on thick slices but the model cannot perform well on thin-slice images as shown in the first row of Table 4. However, without the annotation from images, the prediction results would be extremely unreliable since the objective function reached the global minimum when the probability of each class was assigned the same value. Moreover, when incorporating both thick-slice and thin-slice images into the training under the proposed semi-supervised paradigm, our method could result in better performance on thin-slice images by at least 3.5% improvement on the Dice coefficient compared to the model in Exp 1, while it only sacrificed little performance on thick images. The rationale behind this is that our model can learn a shared feature representation for both thick-slice and thin-slice images, which can be beneficial for handling different types of images.

**Table 4:** Dice coefficient comparison for our ablation studies.

**Exp**.	**Thick**	**Thin**	**MRI**			**CT**		
			**Thick**	**Thin**	**Mixed**	**Thick**	**Thin**	**Mixed**
1	√		**0.9390**	0.8199	0.8391	**0.9438**	0.8345	0.8767
2		√	0.0034	0.0108	0.0110	0.0109	0.0006	0.0069
3	√	√	0.9323	**0.9056**	**0.9099**	0.9365	**0.8697**	**0.8954**

*Trained our model with different thickness images. “Thick” represents our model is trained with labeled thick-slice images, and “Thin” means the model is trained with thin-slice images without annotations. We can see with the help of the semi-supervised training technique we proposed, our method can gain a significant improvement on thin-slice images. Bold values indicate the best performance*.

### Qualitative Analysis

**Figure 3:**
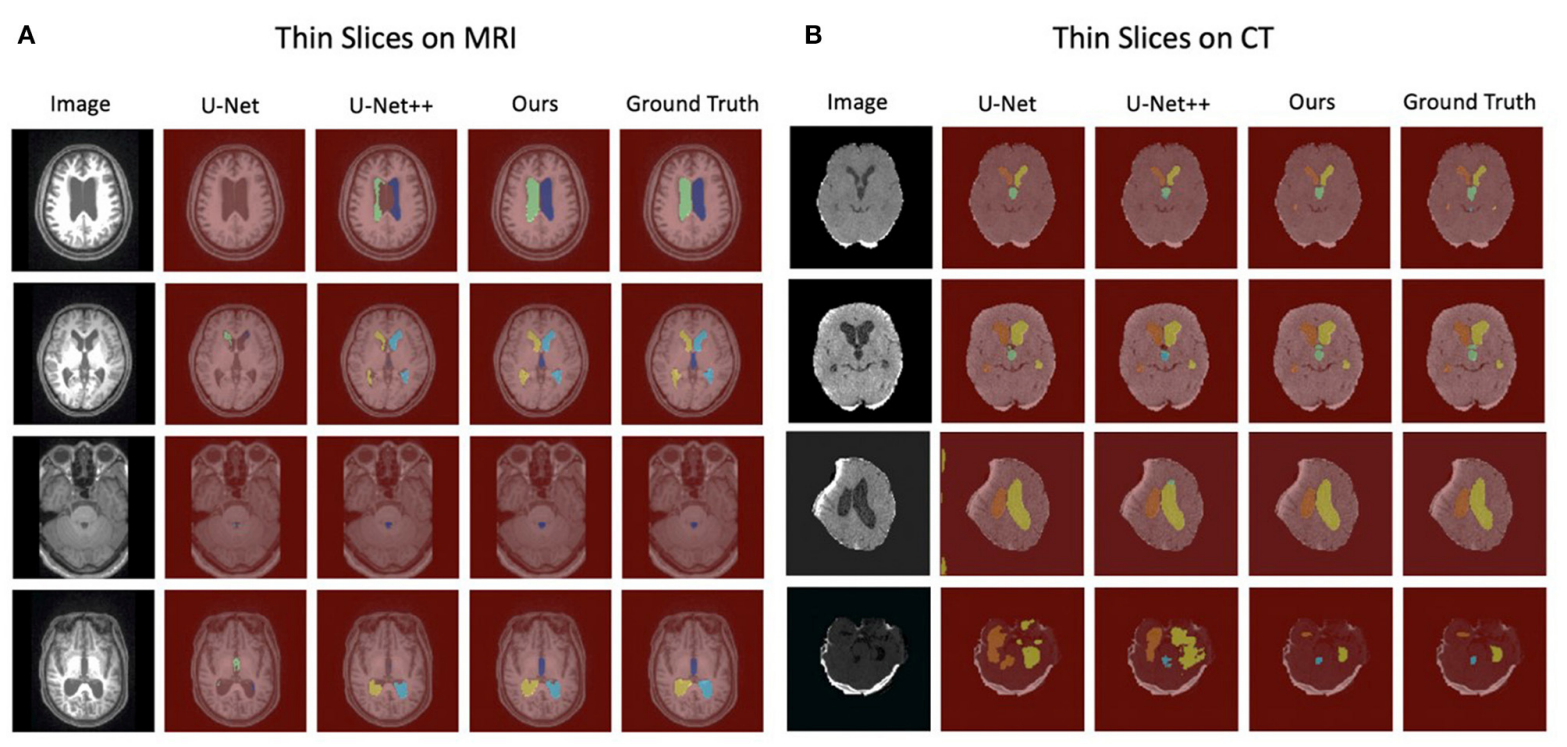
The visualization of segmentation results for thin-slice with MRI images and CT images. **(A)** The completeness of segmentation indicates the performance of each model on MRI images in which our method achieves the best. **(B)** Our method is superior to other competing approaches on CT images, specifically for low contrast images (The last row).

Figure 3 shows the example segmentation results on randomly selected thin-slice images from the testing set for both MRI and CT modalities. In the second column of each modality, it can be observed that U-Net performed poorly on MRI images. Meanwhile, U-Net++, which is the updated version of U-Net, showed better predictions compared to the U-Net while they were still not accurate. Compared with the U-Net and U-Net++, our method achieved accurate results and could segment both MRI and CT images with high precision. Particularly, in the last row of the CT example, although the original image had low contrast, our method was still able to recognize each part and segmented the data accurately, which has demonstrated the robustness of our method. Divide patients with acquired hydrocephalus into Subarachnoid hemorrhage group, brain trauma group, and brain tumor group according to the cause of the disease. Using our method to automatically segment the images of the three groups of patients, the results of CT images show that the Dice of the three groups are 0.94, 0.95, and 0.94, respectively. The results of the MRI image showed that the Dice of the three groups were 0.91, 0.89, 0.92, respectively (Table 5).

**Table 5:** Thick-slice image segmentation results(Dice) of acquired hydrocephalus.

**Method**	**CT**			**MRI**		
	**Subarachnoid hemorrhage**	**Trauma**	**Tumor**	**Subarachnoid hemorrhage**	**Trauma**	**Tumor**
	**(*n* = 24)**	**(*n* = 13)**	**(*n* = 3)**	**(*n* = 24)**	**(*n* = 10)**	**(*n* = 4)**
U-Net	0.9091	0.9143	**0.9387**	0.9085	0.8729	0.92
U-Net++	0.8903	0.8835	0.9219	0.9004	0.8649	**0.9274**
Ours	**0.9407**	**0.9454**	0.9364	**0.9105**	**0.8919**	0.9231

*Divide patients with acquired hydrocephalus into cerebral hemorrhage group, brain trauma group, and brain tumor group according to the cause of the disease. Using our method to automatically segment the images of the three groups of patients, the results of CT images show that the Dice of the three groups are 0.94, 0.95, and 0.94, respectively. The results of the MRI image showed that the Dice of the three groups were 0.91, 0.89, 0.92, respectively. Bold values indicate the best performance*.

## Discussion

Through Figures 2, 3 and Tables 2–4, we can observe that our automated ventricle segmentation method can be successfully applied CT and MRI images with different thicknesses. More importantly, the segmentation results obtained are better (Dice > 0.9) compared to widely used U-Net and its advanced version U-Net++. There is no doubt that the proposed method is promising for different clinical scenarios.

### Clinical Significance of the Automated Ventricle Segmentation

Changes in the shape and size of the ventricles are associated with many diseases, and relevance ventricular enlargement is a crucial marker of brain atrophy associated with normal or pathological aging processes (Schoemaker et al., 2019). Ventricular enlargement also represents a feasible short-term marker of disease progression in mild cognitive impairment and Alzheimer's disease (Nestor et al., 2008). Ventricular enlargement can occur early in the course of Parkinson's disease and is associated with cognitive decline (Apostolova et al., 2012). New/enlarging T2w lesions adjacent to the ventricle wall and thalamic atrophy are independently associated with lateral ventricular enlargement in multiple sclerosis (Sinnecker et al., 2020). Ventricular enlargement is also arguably the most consistent neuroanatomical biomarker present in schizophrenia (Sayo et al., 2012). Whether it is to detect early disease (Dalaker et al., 2011), evaluate the condition of the patient (Ferrarini et al., 2008), diagnose the disease (Relkin et al., 2005), evaluate the effect of surgery (Neikter et al., 2020) or other aspects, accurate measurement of the size of the ventricles has very important clinical significance (Shi et al., 2015).

When following patients with hydrocephalus, the timing for intervention is difficult to decide for clinicians. Therefore, by providing clinicians with an accurate measure of increased ventricle volume, automated ventricular segmentation techniques would give them more information to make their decisions (Qiu et al., 2015). The segmentation of ventricles provides quantitative measures on the changes of ventricles in the brain that form vital diagnostics information (Chen et al., 2009). The automated segmentation of ventricles can assist in making a differential diagnosis of ischemic stroke. The quantitative measurement of the ventricles can be helpful in a treatment, recovery, and follow-up process. The segmented ventricles can also serve as the reference in determining the spatial position of the infarct, which can provide useful information for treatment planning (Poh et al., 2012). Accurate and automated segmentation and labeling tools enable more sophisticated evaluations of the ventricular system in neurodegenerative diseases, cerebrospinal fluid disorders, as well as in normal aging (Shao et al., 2019). Various diseases affect the size and morphology of the ventricles, and knowledge of the normal and abnormal ventricular system is essential in understanding various pathological states. For these reasons, it is critical to extract the ventricular system to ascertain its morphology and volume (Xia et al., 2004). By manually labeling the ventricles, the time required to measure the volume and relative ventricle volume of each subject is about 30 min, which is acceptable in research, but obviously not feasible in clinical practice (Ambarki et al., 2010). Using the automatic ventricle segmentation method can save time significantly. Besides, the unsupervised segmentation method can leverage the dependency of labeled data which is more practical for a real-world scenario. For instance, Liu et al. (2019) utilized the quality of merged segmentation results to update the ensemble weights of different segmentation results in order to achieve accurate segmentation results. Meanwhile, Ganaye et al. (2018) took advantage of the invariant nature of the anatomical structure to improve the robustness of the segmentation results by applying semantic constraints.

### Comparison Studies and the Advantages of Our Proposed Method

Huff et al. mentioned the limitations of their research for an automated ventricle segmentation method that all the studies were performed on similar CT scanners with similar acquisition parameters and identical slice thicknesses. No pathological ventricles were included in this study other than simply enlarged ventricles (Huff et al., 2019). In our research, we can see that data acquired from different scanners were validated, the thickness of the scan layer was also different, and pathological ventricles were also included. Qiu et al. (2015) outlined the limitations of their study as validated on a limited number of images since MR images of preterm neonates were usually not performed at our center unless a severe disease was suspected. Because of this, we did not deliberately make requests when selecting patients. As our goal is to automatically segment the ventricles, the image we chose must be systematic and comprehensive. The shape and size of the ventricles of the four groups of patients can represent the ventricles of the elderly cohort. Kocaman et al. (2019) performed a study on a small number of individuals. The actual sample size of our four groups of patients is large, and in clinical practice, these patients are relatively common. As the sample size increased, the results of our automated ventricle segmentation method were also gradually stabilized.

Xia et al. pointed out the limitations of their research on automated extraction of the ventricular system: When the slice thickness, especially in coronal and axial directions, is too high, the algorithm could not work satisfactorily. Most of the subjects tested did not have any pathology or major distortion of the ventricles (Xia et al., 2004). In our research, the thickness of the scan slice was no longer a confounding factor. Both thick-slice and thin-slice images could be processed with better ventricle segmentation results. At the same time, our patients included not only normal elderly people but also brain atrophy elderly people with slight changes in the ventricle shape and size. Besides, our proposed framework also performed well for iNPH patients with significant changes in the shape and size of the ventricles. More importantly, the elderly with acquired hydrocephalus with obvious changes in intracranial structures caused by trauma, tumor, hemorrhage, and other conditions were also included. Shao et al. mentioned in their brain ventricle parcellation work that the proposed network was also robust to white matter hyperintensities (WMH), which were often associated with NPH and located adjacent to the lateral ventricles. WMH can sometimes negatively affect the outcome of automated segmentation algorithms (Shao et al., 2019). Similarly, some of our patients were also NPH patients, and they had white matter hyperintensities around their lateral ventricles. Some patients with brain atrophy had a similar situation, but our ventricle segmentation framework could handle it.

### Influence of Examination Type and Slice Thickness on Ventricle Segmentation

In clinical work, we often choose CT or MRI for head imaging examination. Both methods have their advantages and disadvantages. For CT, it is relatively convenient to operate, no need to worry about metal implantation, and the inspection speed is fast. But it has ionizing radiation to the human body, and it has a low signal to noise ratio and relatively low contrast. For MRI, it does not produce ionizing radiation and can provide better soft-tissue resolution, but its inspection time is long and may have considerable issues such as metal implantation and claustrophobia and other related problems (Chen et al., 2009; Liu et al., 2010; Coupe et al., 2011; Poh et al., 2012; Qian et al., 2017; Huff et al., 2019). It is well known that in medical images partial volume effect is inevitable. Reducing the slice thickness can reduce the partial volume effect. But for CT examinations, this means that patients have to receive more ionizing radiation, and for MRI examinations, the examination time will be longer. For deep learning, more content means more information, so thin-layer images are naturally the best choice. However, in clinical practice, because of the heavy burden for a large patient population, thick-slice scanning is still the most used acquisition method. But for the segmentation of the ventricles from thick-slice images, the number of images per patient is small, and the information that can be extracted is also limited. Coupled with the influence of the partial volume effect, it is often difficult to segment the boundaries of the ventricles from thick-slice images (Xia et al., 2004). The stroke area on the CT image is often adjacent or connected to the ventricle area, and the grayscale is similar, which increases the difficulty of accurately segmenting the ventricle (Qian et al., 2017). In addition, on the CT image, due to the noise and low contrast between the soft tissues, there is no obvious peak in the cerebrospinal fluid in the whole brain intensity histogram. This makes it difficult to find a suitable threshold for cerebrospinal fluid using traditional histogram-based segmentation methods (Liu et al., 2010). Part of the volume effect will affect the segmentation of the ventricle, especially on MR images with limited resolution (Coupe et al., 2011). Due to the partial volume effect, there exist transition regions between the Cerebrospinal fluid and gray matter, if these transition regions are completely excluded, the ventricular system is under-segmented, and some ventricular components, for example, the lateral ventricles, may be broken into several disconnected parts (Liu et al., 2009). The temporal horns and occipital poles of the ventricle can be separated from the main body. When the shape-based ventricle segmentation method and the ventricle segmentation method based on the regional growth technology are used, the results will be affected. In addition, the signal intensity of the choroidal plexus is similar to that of gray matter. When a simple threshold technique is used to segment the ventricle, the result will also be affected (Coupe et al., 2011). All in all, different imaging data and slice thickness have their advantages and disadvantages, and they also have a different impact on automated segmentation methods.

### Limitations of Our Automated Ventricle Segmentation Framework

Because our current work was a retrospective study based on the elderly to establish a new systematic automated ventricle segmentation method. Therefore, our research might still have some limitations. First of all, because this study selected elderly patients, our method might have insufficient capacity to deal with pediatric patients. Secondly, because this was multi-center and multi-modal research, in terms of results, our goal was to perform well as a whole, so the expression of results in some respects was bound to be relatively weakened. When processing cross-hospital data, we need to handle extensive re-training of the model to ensure the accuracy of the running results. As a deep learning based model, the training data collected at one site are often unavailable to others due to privacy and legal issues (Wang et al., 2020a,b).

In future research, we will focus on extracting different imaging and biological features through deep learning of images, laboratory test results, and clinical information of patients with abnormal ventricles. We will achieve a systematic and comprehensive analysis of patients with ventricular abnormalities, and determine whether the patient has a certain disease that can cause ventricular abnormalities.

## Conclusion

In order to systematically and comprehensively assess the size of the ventricle of elderly patients, we have established an automated ventricle segmentation method. This automated ventricle segmentation method can not only be applied to both CT and MRI images but can be also applied to images with different slice thicknesses. More importantly, it produces superior segmentation results. Deploying this automated ventricle segmentation method in the clinical scenarios can help doctors to find and diagnose early disease, evaluate the progress of the patient's condition, and inform the treatment planning for the patients. At the same time, the medical image scanning method and the slice thickness are no longer limitations for automated ventricle segmentation. There is no doubt that the proposed method will have a wide application in clinical studies.

## Data Availability Statement

The original contributions presented in the study are included in the article/supplementary material, further inquiries can be directed to the corresponding author/s.

## Ethics Statement

The studies involving human participants were reviewed and approved by Ethics Committee of The First Affiliated Hospital of Shenzhen University and Shenzhen Second People's hospital. Written informed consent for participation was not required for this study in accordance with the national legislation and the institutional requirements.

## Author Contributions

XZ, QY, YJ, JX, and GY conceived and designed the study, contributed to data analysis, contributed to data interpretation, and contributed to the writing of the report. XZ, ZN, WM-S, EF, ZL, and JX contributed to the literature search. XZ and JX contributed to data collection. XZ, QY, YJ, and MW performed data curation and contributed to the tables and figures. All authors contributed to the article and approved the submitted version.

## Conflict of Interest

ZN and WM-S are employed by Aladdin Healthcare Technologies Ltd. QY, YJ, and MW are employed by Hangzhou Ocean's Smart Boya Co., Ltd., China and Mind Rank Ltd., China. The remaining authors declare that the research was conducted in the absence of any commercial or financial relationships that could be construed as a potential conflict of interest.
